# Role of transition metal exporters in virulence: the example of *Neisseria meningitidis*

**DOI:** 10.3389/fcimb.2013.00102

**Published:** 2013-12-23

**Authors:** Cyril Guilhen, Muhamed-Kheir Taha, Frédéric J. Veyrier

**Affiliations:** Département Infection et Epidémiologie, Institut Pasteur, Unité des Infections Bactériennes InvasivesParis, France

**Keywords:** virulence factors, *Neisseria meningitidis*, metals, exporter, efflux

## Abstract

Transition metals such as iron, manganese, and zinc are essential micronutrients for bacteria. However, at high concentration, they can generate non-functional proteins or toxic compounds. Metal metabolism is therefore regulated to prevent shortage or overload, both of which can impair cell survival. In addition, equilibrium among these metals has to be tightly controlled to avoid molecular replacement in the active site of enzymes. Bacteria must actively maintain intracellular metal concentrations to meet physiological needs within the context of the local environment. When intracellular buffering capacity is reached, they rely primarily on membrane-localized exporters to maintain metal homeostasis. Recently, several groups have characterized new export systems and emphasized their importance in the virulence of several pathogens. This article discusses the role of export systems as general virulence determinants. Furthermore, it highlights the contribution of these exporters in pathogens emergence with emphasis on the human nasopharyngeal colonizer *Neisseria meningitidis*.

## Introduction

For decades, it has been known that transition metals played a role in regulating host pathogen relationships (Weinberg, 1971; Finkelstein et al., 1983). Bacterial pathogens must acquire these metals in order to survive in the host during an infection. Metals such as Fe, Mn, Zn, Ni, Cu, Co, and Mo (Schaible and Kaufmann, 2005) have an incomplete “d” orbital which permits different states of oxidation, e.g., Fe^2+^ and Fe^3+^. These metals often serve essential roles in protein structural stabilization or as enzymes cofactors (Barondeau and Getzoff, 2004). However, the unique chemistry of these metals can also provoke inappropriate redox reactions with O^−^_2_ and H_2_O_2_, (Fenton's reaction), generating highly damaging hydroxyl radicals (OH and OH^−^) that can ultimately lead to the cell death (Stadtman, 1990). It is perhaps this duality that has driven the selection of sophisticated bacterial strategies to orchestrate transition metal homeostasis by sensing, acquiring, storing, or when necessary, exporting these essential but potentially lethal metals.

The stringent and complex requirements of bacterial pathogens for metals have been exploited by the immune system to limit bacterial growth. The majority of described examples demonstrate that the immune system uses starvation strategies that consist in decreasing metal availability (mainly Fe^2+^, Zn^2+^, and Mn^2+^) (Canonne-Hergaux et al., 1999; Corbin et al., 2008) to restrict bacterial growth. However, recent reports suggest the existence of an immune strategy whereby the bacteria are poisoned with an overload of metal, principally Zn^2+^ and Cu^+/2+^ (White et al., 2009). This latter finding also corroborates the fact that deletion of bacterial efflux pumps often impairs the virulence of pathogens (Stahler et al., 2006; Rosch et al., 2009; Botella et al., 2011; Li et al., 2011; Veyrier et al., 2011; Padilla-Benavides et al., 2013). It is therefore important to call attention to these efflux systems as virulence factors, as they have received less attention than metal importer systems. This article offers a brief perspective of the different families of metallo-exporters and a discussion of their general importance in the virulence of bacterial pathogens with emphasis on *Neisseria meningitidis*, an obligate human respiratory symbiont.

## Metal efflux systems

**Figure 1:**
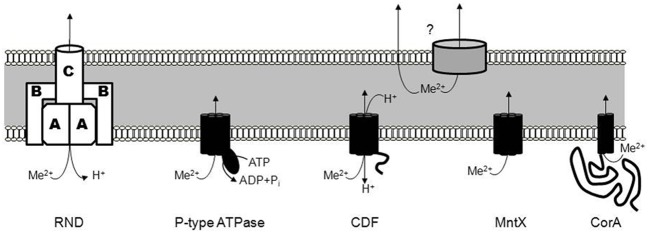
**The different families of metal exporters.** The different families of metal exporters include the Resistance-Nodulation-Cell division (RND) type transporters, the P-type ATPase family (forming a covalent phosphorylated intermediate), the Cation Diffusion Facilitator (CDF) family, the MntX (Transporter Mediating Manganese Export) family, and the CorA family (Cobalt Resistance protein A). As schematized by a question-mark, the subsequent export from the periplasm to the extracellular space can be mediated via an unknown porin or passively.

The concentrations of metals can vary dramatically in the host organism during the course of a bacterial infection, and pathogens have developed a large panel of exporters to regulate their intracellular metal concentrations. Currently five main classes of bacterial exporters (Figure 1) have been described:

1. The Resistance-Nodulation-Cell division (RND) type transporters are integral membrane proteins mediating the efflux of a broad variety of substrates with a subset exporting metals. This subgroup is named heavy-metal efflux RND (HME-RND). This tripartite transporter utilizes the proton motive force to drive the efflux of the substrates (Nies, 1995; Goldberg et al., 1999). The RND pump (annotated A in Figure 1) is an integral membrane protein with a hydrophilic periplasmic component, connected to a trimeric outer membrane factor (C in Figure 1). This outer membrane channel allows the efflux of metal into the extracellular space (Paulsen et al., 1997). The third part of the RND transporter complex is composed of several units of a periplasmic membrane protein (B in Figure 1). It serves as an adaptor that forms a ring around the outer membrane proteins and the pump, thereby stabilizing contact between the two other components (Murakami et al., 2002; Akama et al., 2004a,b).
  Several RND exporter systems have been identified to date, including CzcABC from *Cupriavidus metallidurans* which mediates the efflux of Co^2+^, Zn^2+^, and Cd^2+^ with different affinities. The deletion of *czcC* (the outer membrane component) resulted in a decrease of Cd^2+^ and Co^2+^ efflux whereas the lack of *czcA* or *czcB*, the pump and fusion protein, respectively, induced a complete loss of efflux activity (Nies and Silver, 1989; Nies et al., 1989; Goldberg et al., 1999). Other characterized RND systems include CnrABC from *Alcaligenes eutrophus* which mediates the efflux of Co^2+^ and Ni^2+^ (Liesegang et al., 1993), CznABC from *Helicobacter pylori* which mediates resistance against high concentrations of Ni^2+^, Zn^2+^, and Cd^2+^ (Stahler et al., 2006) and CusABC from *E. coli* which mediates the efflux of Cu^+^ and Ag^+^ (Long et al., 2012).
2. Members of the P-type ATPase family (Figure 1) are present in eukaryotes and prokaryotes and, as the name implies, they couple metal transport to the hydrolysis of ATP (Fagan and Saier, 1994). Catalytic phosphorylation of the transporter occurs after binding of cytoplasmic metal to the trans-membrane metal-binding sites. This phosphorylation is reported to induce a permissive conformation allowing the translocation of metals (Stokes et al., 1999). Two substrate classes have been defined for the family: Zn^2+^/Cd^2+^/Pb^2+^ or Cu^+^/Ag^+^. Exporters specific for the former class include ZntA from *E. coli* (Rensing et al., 1997), CadA from *Bacillus subtilis* (Solovieva and Entian, 2002; Gaballa and Helmann, 2003), and CtpC from *Mycobacterium tuberculosis* (Botella et al., 2011). The second class is exported by homologs of CopA from *Streptococcus pneumoniae* (Shafeeq et al., 2011). CopA, the Cu^+^-efflux P-type ATPase, maintains a low cytoplasmic copper concentration in conjunction with other members of a single operon encoding the Cu^+^-dependent repressor, CopY (Portmann et al., 2006) and CupA, a cell membrane-anchored Cu^+^-chaperone (Fu et al., 2013). Biochemical characterization of the CopA exporter indicates an eight transmembrane-helix topology forming a Cu^+^ transport channel (Padilla-Benavides et al., 2013).
3. An important and diverse class of metal exporters is the Cation Diffusion Facilitator (CDF) family (Figure 1). CDF members transport various metals in both prokaryotes and eukaryotes (Haney et al., 2005). The first description of a CDF protein was YiiP from *E. coli* and its function, the efflux of Cd^2+^ and Zn^2+^, is coupled to H^+^ antiport. In addition, Fe^2+^ was also suggested to be exported through YiiP (Grass et al., 2005) but subsequent studies have shown that Fe^2+^ transport is not as efficient as for Cd^2+^ and Zn^2+^ (Wei and Fu, 2005; Hoch et al., 2012). YiiP and other members of the family are usually composed of six transmembrane domains followed by a metallochaperone-like cytoplasmic domain that regulates metal transport activity (Lu et al., 2009). Crystal structures revealed an inward-facing homodimeric structure with four Zn^2+^ binding sites per monomer, designated Z1–Z4 (Lu and Fu, 2007; Coudray et al., 2013). Several homologs with specificity for Zn^2+^ have been described, including ZitB in *E. coli* (Chao and Fu, 2004), ZitA in *M. tuberculosis* (Nies, 2003), CzcD in *B. subtilis* (Guffanti et al., 2002) or *S. pneumoniae* (Kloosterman et al., 2007).
  A subclass of CDF exporters, MntE, with a preference for Mn^2+^ has also been described. In general Mn has been viewed as completely beneficial for the bacteria. For instance, it has been established that Mn plays a role in resistance to superoxide and hydrogen peroxide in several bacteria including *S. pneumoniae* (Yesilkaya et al., 2000; McAllister et al., 2004) *Bradyrhizobium japonicum* (Hohle and O'Brian, 2012) and *Neisseria gonorrhoeae* (Seib et al., 2006) among others. Accordingly Mn^2+^ importers, e.g., MntH and MntABC, have received the majority of attention until recently, when MntE-dependant Mn^2+^ export was described in *S. pneumoniae* (Rosch et al., 2009).
4. Several recent studies have also shown that MntE is not the only type of Mn^2+^ exporter and the existence of a new and distinct family corresponding to the fourth type of exporters, was revealed: the MntX (Transporter Mediating Manganese Export) family (Figure 1) (Li et al., 2011; Veyrier et al., 2011). Little is known about the MntX family transport mechanism but secondary structure and topological predictions suggest an inverted repeat of three transmembrane segments, i.e., DUF204 (Veyrier et al., 2011). Unlike the other families, MntX is found exclusively in the bacterial kingdom, indicating the family may have a relatively recent origin following the genetic fusion of two DUF204 domains (Veyrier et al., 2011). For the moment, homologs of MntX in *N. meningitidis* (Veyrier et al., 2011), *Xanthomonas* sp. (Li et al., 2011; Veyrier et al., 2011), and *E. coli* (Waters et al., 2011) have all been described to export principally Mn^2+^ with some secondary affinity for other divalent metals.
5. The fifth exporter family is composed of a subclass of the 2-TM-GxN family (CorA, Cobalt Resistance protein A) that was first identified as Mg^2+^ transporters (Figure 1) (Smith et al., 1993). However, some members are dedicated to the export of other divalent cations, principally Zn^2+^. ZntB of *Salmonella enterica* is involved in the transmembrane flux of Zn^2+^ and Cd^2+^ (Worlock and Smith, 2002). This protein, which may multimerize, harbors two transmembrane domains and a long cytoplasmic region that facilitates acquisition and subsequent delivery of cations to the transport channel.

## Metal efflux and virulence

As stated before, metal chelation is used by the immune-system to restrict bacterial growth and, by definition, the use of exporters may not help the bacteria in this situation. From the above description, the majority of exporters from bacterial pathogens are dedicated to export of three transition metals (Zn, Cu, and Mn) that are found in substantial amount in the human body. Interestingly, Zn^2+^ levels are increased during inflammation (20) and have long been recognized to regulate the immune system (13). In addition, Zn^2+^ is also present in phagosomes containing bacteria (35). Thus, it is postulated that pathogenic bacteria uses Zn^2+^ export systems to face the fluctuating levels of Zn^2+^ during infection of the human body. The same may apply for copper, as it has been recently demonstrated that animals use copper as an anti-microbial weapon by inducing oxidative stress (26). Similarly, Cu^+^ is imported into the phagosome via the protein ATP7A (White et al., 2009). It is therefore not surprising that pathogens lacking Zn^2+^ or Cu^+^ exporters have impaired virulence. As an example, it has been established that a *copA* mutant strain showed decreased virulence in a mouse model of pneumococcal pneumonia and a decreased ability to survive in the mouse nasopharynx (NP), indicating that Cu^+^ homeostasis plays an important role in *S. pneumoniae* physiology and virulence (26). All together, these studies suggest that both metals are used by the immune system to intoxicate bacteria (Botella et al., 2012).

The role of Mn^2+^ during infection is less understood as both beneficial and adverse effects have been reported. Nevertheless, all studies point to a general but important role of Mn^2+^ export in bacterial pathogenesis. All the pathogens tested to date have a decrease in their virulence when deprived of their Mn^2+^-exporters. In *S. pneumonia*e, it was demonstrated that the lack of MntE, belonging to the CDF family, reduced virulence by diminishing both nasal colonization and blood invasion, resulting in decreased mouse mortality (Rosch et al., 2009). The same applies to *Xanthomonas oryzae* pv. *oryzae* in a plant model of infection or *N. meningitidis* in a mouse sepsis model of infection after inactivation of their Mn^2+^ exporter from the MntX family (Li et al., 2011; Veyrier et al., 2011). These data could suggest the existence of a host immune strategy based on Mn^2+^ poisoning. This hypothesis is somewhat discordant with the recent description of a host Mn^2+^ and Zn^2+^ chelator, calprotectin (Corbin et al., 2008). This host factor is capable of inhibiting bacterial growth in a Mn^2+^-dependent manner (Damo et al., 2013), thereby fulfilling an important role in the protection of the host against infection by bacterial pathogens such as *S. aureus* (Corbin et al., 2008; Damo et al., 2013). Furthermore, the poisoning hypothesis is also in disagreement with the fact that deletion of Mn^2+^ importers decreases the virulence of several pathogens (Boyer et al., 2002; Anderson et al., 2009; Champion et al., 2011; Perry et al., 2012). For this reason, further study of this phenomenon will be important to understand the exact role that Mn^2+^ plays during infection (poison, nutrient or both).

## Metal export: the example of *N. meningitidis*

The NP defines the upper part of the pharynx from the end of nasal cavities (choanoe) to the upper surface of the soft palate. On the lateral parts it communicates with the Eustachian tubes by the pharyngeal ostium whereas the posterior part is composed of the pharyngeal tonsils (adenoids). This compartment is open and serves as a habitat for many microorganisms which are collectively called the NP microbiota (or flora). In this sense, the NP is the ecological niche for many bacterial pathogens such as *N. meningitidis*, *S. pneumoniae*, *Haemophilus influenzae*, and *Moraxella catarrhalis*. While carriage is usually asymptomatic, it can occasionally evolve into local infections of the upper-respiratory tract (pharyngitis, laryngitis, bronchitis, sinusitis, and otitis) or an invasive infection leading to life threatening diseases, such as invasive pneumonia, septicemia, and meningitis. Consequently, this leads to major morbidity and mortality as well as public health and economic burdens.

*N. meningitidis* is exclusively found in humans and frequently isolated from the upper respiratory tract of asymptomatic carriers (overall 10% of the general population). It is also the causative agent of life threatening invasive infections such as septicemia and meningitidis. The carriage of *N. meningitidis* is low in children (around 4–7%) where the principal neisserial colonizer (around 15%, Cartwright et al., 1987) is a closely related, non-pathogenic species, *Neisseria lactamica*. The prevalence of *N. meningitidis* increases after 10 years old with a peak at 19 years old (around 24%) and decreases throughout adulthood (13% in 30-year old to 8% in 50-year old) (Christensen et al., 2011).

The importance of Fe import systems for *N. meningitidis* virulence has been previously demonstrated following deletion of genes coding for several transporters (Genco et al., 1991; Genco and Desai, 1996; Larson et al., 2002; Renauld-Mongenie et al., 2004; Hagen and Cornelissen, 2006). A complementary approach has been used in which its virulence was enhanced by providing a compatible Fe source (Oftung et al., 1999; Zarantonelli et al., 2007). The role of exporters in the virulence of this nasopharyngeal pathogen is less-well established. The emergence of data concerning the role of exporters as virulence determinants, and the new concept of bacterial metallo-intoxication by the immune system, should encourage future research on this topic.

Our group has recently identified a novel Mn^2+^-exporter, MntX. We showed the *mntX* gene is expressed during sepsis in a mouse model and required for full virulence (Veyrier et al., 2011). We have further shown that MntX is required to maintain the Fe/Mn ratio thus avoiding molecular replacement between these two metals (Veyrier et al., 2011). Meningococci acquire Fe from host sources such as albumin, transferrin, or Fe-citrate which have also been shown to bind Mn. Therefore, a portion of these molecules is complexed with Mn in the host (concentration in the μM range) (Michalke et al., 2007). We speculate, based on the well-described needs of *N. meningitidis* for Fe (Genco et al., 1991; Genco and Desai, 1996; Larson et al., 2002; Renauld-Mongenie et al., 2004; Hagen and Cornelissen, 2006), that the intensive import of Fe could result in non-specific import of other divalent metals, which consequently must be exported. In this sense, we observed a specific Mn^2+^-export by MntX of *N. meningitidis* whilst the homologous exporter of *X. campestris* was also able to export Fe^2+^ to some extent (Veyrier et al., 2011). In this case, metallo-exporters may be required to maintain the optimal ratio between different metals (e.g., Mn/Fe and Mn/Zn). Non-specific metal uptake should be also considered as a complementary hypothesis to Mn-intoxication by the host immune system. As another alternative, it has been reported that *N. meningitidis* harbors a specific Mn-dependant hemolysin called HrpA (Michalke et al., 2007). It is therefore possible that MntX (and other exporters) delivers Mn^2+^ to *N. meningitidis*-specific extracellular virulence factors such as HrpA.

Although *N. gonorrhoeae* is closely related to *N. meningitidis* it generally resides asymptomatically in the female genitourinary tract. This ecosystem is more anaerobic than the NP and is also occupied by H_2_O_2_ producing lactobacilli. These features of the genitourinary tract are known to increase bacterial requirement for intracellular Mn^2+^ and that may explained the high proportion of strains of *N. gonorrhoeae* harboring a premature stop codon mutation in the *mntX* gene (Veyrier et al., 2011).

## Metal efflux systems and the evolution of *N. meningitidis*

**Figure 2:**
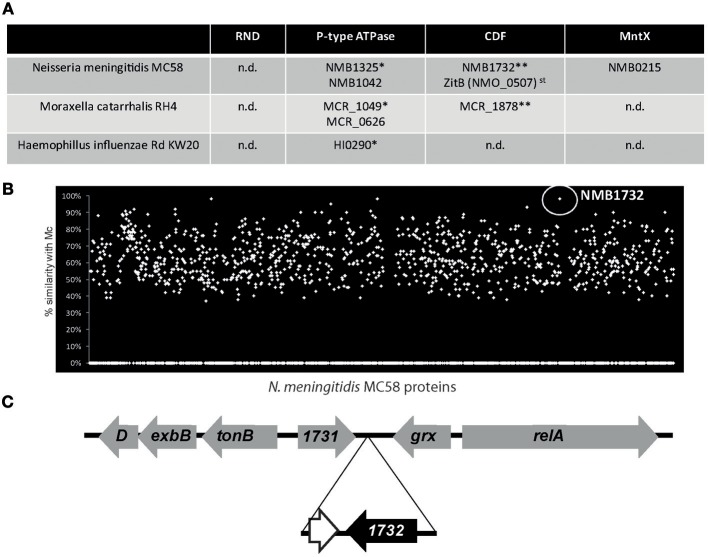
**The example of *Neisseria meningitidis* exporters. (A)** Candidate for metal exporters encoded in the genomes of three nasopharyngeal pathogens based on the Kyoto Encyclopedia of Genes and Genomes (KEGG database). st stands for strain specific and n.d. for not detected. ^*^ and ^**^ represents groups of putative homologous genes determined using blastp similarity. **(B)** TBlastN of *N. meningitidis* MC58 proteins against the genome of *M. catarrhalis* RH4 (Mc). **(C)** Gene organization of the NMB1732 locus. The genes in gray have common organization between *N. meningitidis* and *N. gonorrhoeae* whereas the genes in black are *N. meningitidis* specific. The white gene, adjacent to NMB1732, corresponds to a remnant of DNA methyltransferase also present in the genome of *M. catarrhalis*.

The discovery of MntX highlights the importance of metal efflux system in the virulence of *N. meningitidis*. Importantly, the genome of *N. meningitidis* harbors other putative metal exporters. Figure 2A presents the genes with homologies to putative exporters detected in the genomes of *N. meningitidis* and two other major Gram-negative pathogens of the NP: *M. catarrhalis* and *H. influenzae*. Although the genomes have similar sizes, *N. meningitidis* seems to harbor more efflux systems. Only one gene was common to all three species, NMB1325, which shares a high similarity with HI0290 (81%) and with MCR_1049 (68%). As Fe importers are often present in horizontally transferred pathogenicity islands, we wondered if this could be the case for additional metallo-exporters, and if some of them could have been specifically acquired by *N. meningitidis*. As this bacterium is a human specific nasopharyngeal pathogen, without possibility of survival in the external environment, acquisition of such exporter by horizontal gene transfer (HGT) would support a role of these exporters in the emergence of *N. meningitidis*.

With the exception of NMB1732, all the putative exporters identified have homologs in other *Neisseria* species, and therefore cannot represent examples of recent HGT. NMB1732 is rarely present (if ever) in the genomes of other *Neisseria* species (such as *N. gonorrhoeae*) nor in other closely related non-neisseria strains (e.g., *Kingella*). In addition, all the isolates of *N. meningitidis* sequenced to date (~200) harbor this gene, coding for a protein from the CDF family. As a consequence, this *N. meningitidis* specific gene is used by the Centre National de Reference des Meningocoques (CNRM) that is located within our laboratory, at the Institut Pasteur in Paris, to definitively distinguish *N. meningitidis* from all the other *Neisseria* species by PCR. Surprisingly, this gene and the surrounding region have an unusually high identity (99%) with a region of the genome of the non-closely related *M. catarrhalis* (Figure 2B) which shares the same ecosystem. Moreover, the gene is also present in other *Moraxella* species. Altogether, these findings suggest a possible transfer of the CDF exporter NMB1732 from *M. catarrhalis* to *N. meningitidis*. Our hypothesis is reinforced by the fact that, with the exception of NMB1732 and the adjacent pseudogene, the organization of the locus (including *tonB*) is conserved between *N. meningitidis* and other closely related *Neisseria* species (Figure 2C). The importance of MntX for *N. meningitidis* virulence and the acquisition by HGT and the conservation of NMB1732, a putative CDF exporter, highlight the pathogen's needs for metal exporters and the role that these exporters have played in the emergence of pathogens.

## Conclusion

The existence of exporters and their transfer between species has been known for a long time in the context of bacterial living in the environment. In the last few years, bacterial metallo-exporters have also been demonstrated to play a role in the context of infection. The immune system uses Cu^+/2+^ or Zn^2+^ to poison bacteria and export systems are used to detoxify this overload. The role of Mn is not yet completely understood, but MntE and MntX are the proof of concept that Mn^2+^ exporters are important for pathogenesis. Nevertheless, further research is required to understand the role of exporters in emergence and adaptation of pathogens and how these efflux systems can be used to thwart the host immune system defenses.

### Conflict of interest statement

The authors declare that the research was conducted in the absence of any commercial or financial relationships that could be construed as a potential conflict of interest.
